# Oxygen efficient respiratory Aid (OxEra^TM^) device: A safety study

**DOI:** 10.1016/j.afjem.2022.03.003

**Published:** 2022-05-20

**Authors:** Midhun Thomas John, Sarah Alexandra van Blydenstein, Shahed Omar, Joanne Bruins, Stephilia Tshukutsoane

**Affiliations:** a Internal Medicine Registrar, Chris Hani Baragwanath Hospital, Internal Medicine, University of Witwatersrand, South Africa; b Specialist Pulmonologist, Chris Hani Baragwanath Hospital, Internal Medicine, University of Witwatersrand, South Africa; c Critical Care Physician, Chris Hani Baragwanath Hospital, ICU Department, University of Witwatersrand, South Africa; d Chris Hani Baragwanath Hospital, Internal Medicine, University of Witwatersrand, South Africa; e Burns Hons, Critical Care Nurse, RM, RN. Chris Hani Baragwanath Academic Hospital, South Africa

**Keywords:** Safety study, OxEra, Oxygen delivery device

## Abstract

**Background:**

Severe Coronavirus Disease 2019 (COVID-19) can develop pneumonia with severe complications. The Oxygen Efficient Respiratory Aid (OxEra^TM^) device has been granted SAPHRA approval for emergency COVID-19 pandemic use. The device has the potential to be used widely in the healthcare sector due to its efficient oxygen supply and adjustable wall positive expiratory pressure (PEP).

**Objectives:**

We assessed whether the OxEra^TM^ device was safe to use in a healthy adult volunteer population. Our primary objective was to ensure there was no asphyxiation, as assessed by changes observed from baseline End Tidal Carbon Dioxide (ETCO_2_) exceeding 6.3 mmHg and above the 45 mmHg threshold. We also monitored changes in vital organ signs and assessed the pain and comfort of the participant at various intervals with changes in PEPs.

**Methods:**

This was an experimental safety study of the OxEra^TM^ Device on 30 healthy participants at the ICU training centre of Chris Hani Baragwanath Academic Hospital, Johannesburg, South Africa. Each participant had basic vital-signs, ETCO_2_, and Oxygen saturation percentages (SpO_2_%) taken at baseline until the end of 2 h. In the first 20 min, the PEP was increased by 5 cmH_2_0 until 20 min, then continued for the rest of the time on a PEP of 5 cmH_2_0. At each interval, vital signs, subjective comfort, pain, and visual scores were measured.

**Results:**

Thirty healthy participants were enrolled. There was no significant difference in ETCO_2_ from baseline until 2 h. No participant experienced an increase in measured ETCO_2_ greater than 45 mmHg and no increase in ETCO_2_ from baseline was greater than 6.3 mmHg. The median increase in ETCO_2_ over the study period was 2 mmHg. There were no significant changes in respiratory rate and blood pressure. The heart rate decreased significantly (73–68 bpm). The VAS and comfort score had a significant increase over the 2 h from baseline of 0–2 at maximum; however, the PAS scores showed no significant increase.

**Conclusion:**

Overall the OxEra^TM^ device achieved the safety endpoints set out. There was no sign of asphyxiation and there were appropriate physiological responses to changes in PEP once applied. The comfort of the mask did worsen over the 2 h; however, the scores were minimally worse on PEP application but improved once-off PEP. No adverse event was recorded at all.

## African relevance

• Africa is a continent that has many developing countries that have constant strain on their healthcare systems.
• Now with the COVID pandemic, the use of oxygen and need for cost efficient and safe oxygen devices has increased.
• OxEra^TM^ is an oxygen delivery device that has been designed and developed by a consortium named Umoya for emergency use (approval from SAPHRA) in the COVID 19 pandemic.
• Given the potential for the widespread utilisation of this oxygen device in a resource-limited setting, we performed a clinical assessment safety study.

## Introduction

Coronavirus Disease 2019 (COVID 19) is a highly contagious disease caused by a new β coronavirus called Severe Acute Respiratory Syndrome Coronavirus 2 (SARS-COV 2). This new coronavirus is closely related to the 2003 Severe Acute Respiratory Syndrome (SARS) and the 2012 Middle East Respiratory Syndrome (MERS) Coronavirus, of which caused severe respiratory distress, cytokine storm, and severe lung injury [1]. The clinical impact of COVID 19 is mainly on the pulmonary system but has also been documented to affect the extra-pulmonary systems [2].

Non-invasive ventilation has had a successful role in the management of patients with acute hypoxemic respiratory failure [3]. In response to the pandemic, a local volunteer initiative called Umoya collaborated to create the Oxygen Efficient Respiratory Aid ^TM^ (OxEra^TM^) device, which is a simple CPAP device that only requires an oxygen flow rate of 15 L/min. The collaboration included engineers, doctors, designers, 3D printing specialists, and programme managers. The OxEra^TM^ device has received approval by SAPHRA for emergency use in COVID-19 hypoxemic patients. While a multidisciplinary team at the CSIR has evaluated the device relating to the calibration and reliability of the device, there has been no clinical human evaluation.

Most CPAP systems run the risk of inducing rebreathing in case of failure [4]. The OxEra^TM^ device has a built-in safety valve to avoid rebreathing. In the case of rebreathing, a significant rise in ETCO_2_ will occur [5]. This may be accompanied by a decrease in oxygen saturation [5].

Another important challenge related to positive pressure ventilation delivered through a non-invasive device relates to patient comfort and acceptance [6]. The ability of a patient to tolerate the device is therefore vital to the utility of such a device and necessitates the objective assessment of pain and discomfort. Pain and discomfort are notoriously difficult to assess in severe or critically ill patients [7]. Appropriate assessment of pain is key to its management [8]. Tracking physiological parameters including HR and blood pressure together with the use of validated pain tools like the Numerical Rating Scale (NRS) and the Visual Analogue Scale (VAS) are required to objectively assess comfort and pain [9].

Given the obvious potential for the widespread utilisation of this oxygen device in a resource-limited setting we performed a clinical assessment study. To evaluate the safety and patient acceptance of the OxEra^TM^ device, we evaluated the device on a group of healthy volunteers and monitored ETCO_2_, oxygen saturation, physiological variables related to pain, and we performed an objective pain assessment using three different pain and comfort tools.

The aim was to assess the safety and user acceptance of the OxEra^TM^ device using a healthy volunteer population. The primary objective was to monitor for an increase in ETCO_2_ (less than 6.3 mmHg change from baseline ETCO_2_ and no ETCO_2_ above the 45 mmHg threshold). The secondary objective was to monitor changes in vital signs (maintenance of normal pulse oximetry saturation readings (above 93%) and changes in blood pressure, mean arterial pressure, respiratory rate, and HR). The tertiary objective was pain and comfort score assessment (using the OxEra^TM^ at each time interval with varying PEPs).

## Methods

Study Design and Setting: We performed an experimental safety study of the OxEra^TM^ Device at the Intensive Care Unit training centre of the Chris Hani Baragwanath Academic Hospital, Johannesburg, South Africa. This is a large academic facility affiliated with the University of the Witwatersrand. The protocol was approved by the University of Witwatersrand's Human Research Ethics Committee (HREC No: M210222). National Research Database Reference Number GP 202107013. Written informed consent was obtained from each participant (Table 1).

**Table 1:** SpO_2_% readings from baseline to the end of the study.

Variable	Valid N		Minimum	Maximum	25.000thPercentile	75.000thPercentile
	30	100	94	100	97	100
	30	100	96	100	99	100
	30	100	97	100	99	100
	30	100	95	100	99	100
	30	100	98	100	99	100
	30	100	97	100	99	100
	30	100	97	100	99	100
	30	100	98	100	99	100
	30	100	97	100	100	100
	30	100	99	100	100	100

Study population, procedure, and data collection: Thirty healthy adult participants were included in the study. Exclusion criteria included smoking, pregnancy, body mass index (BMI) greater than 30, respiratory and cardiac comorbidities, beards, facial abnormalities, and previous thoracic surgery. An initial safety brief, demonstration, and face mask test to ensure an appropriate seal was followed by the application of the CPAP facemask, an oxygen flow rate of 15 l/min but no positive end-expiratory pressure (PEP = 0). Baseline data (T0) were collected. Data was then collected at the following time points; T1 (5 min, PEP = 5 cmH¬20), T2 (10 min, PEP = 10 cmH¬20), T3 (15 min, PEP = 15 cmH¬20), T4 (20 min, PEP = 20 cmH¬20), T5 (30 min, PEP = 5 cmH¬20), T6 (45 min, PEP =5 cmH¬20), T7 (60 min, PEP = 5 cmH¬20), T8 (90 min, PEP = 5 cmH¬20) and T9 (120 min, PEP = 5 cmH¬20). The data were collected by a trained study Doctor and Nurse. Demographic data of each patient included: height (cm), weight (kg), age (years), race, and gender. The following clinical data were collected at each study time point: ETCO_2_, oxygen saturation percentage, respiratory rate, heart rate, systolic blood pressure, diastolic blood pressure, mean arterial blood pressure, and any adverse events were noted. The visual assessment score (VAS), pain assessment score (PAS), and the comfort score were also taken at each time point. See Appendix I and II for VAS and PAS scores.

Statistical Analysis: All continuous data were described using median and interquartile range (IQR) while categorical data were described using number (n) and percentage (%). Dependent data were compared using the Wilcoxon matched-pairs test (2 groups) or the Friedman ANOVA tests for more than 2 groups. A *p*-value < 0.05 was considered significant.

Sample Size: The sample size was based on the calculation of a single mean estimate. In the case of rebreathing a significant rise in ETCO_2_ will occur [5]. This may be accompanied by a decrease in oxygen saturation [5]. We, therefore, used an ETCO_2_ mean threshold value of 45 mmHg and a standard deviation of 6.3 mmHg. Using a 5% precision, we required a sample size of 30 participants.

Outcomes: The main outcome was to determine if there was a significant increase in ETCO_2_ using a threshold of 45 mmHg or an increment of 6.3 mmHg for the study duration. Secondary and tertiary outcomes included changes in pulse oximetry saturation readings, blood pressure, respiratory rate (RR), HR, and pain and comfort scores for the duration of the study.

## Results

Participant Description: The thirty participants had a median age of 30, with the youngest being 25 years of age and the oldest being 41 years of age. The median weight was 71.5 kg with an interquartile range from 61 to 80 kg. The median height measured was 170 cm with an interquartile range of 167–176 cm. Therefore, the median Body Mass Index (BMI) was 24.7 kg/m^2^, with interquartile ranges of 21.9–25.9 kg/m^2^.

**Fig. 1:**
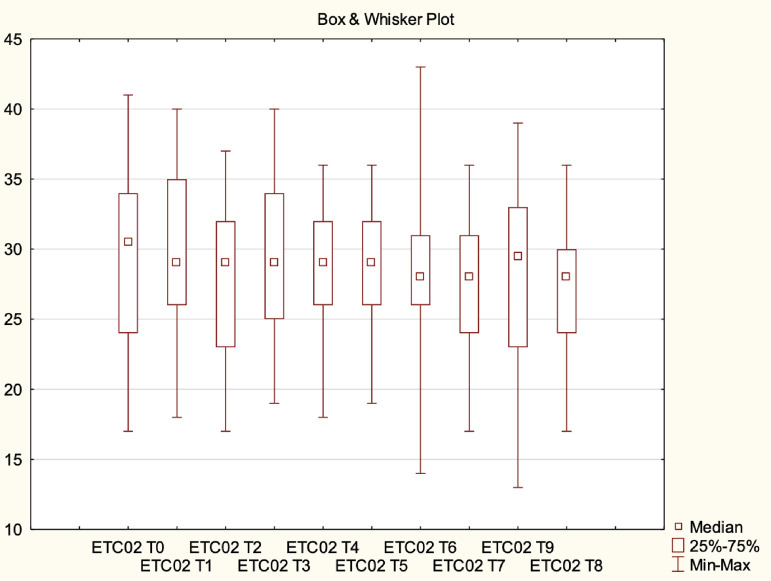
Box and whisker plot of ETCO_2_ from start to end.

Main Outcome: There was no significant difference in ETCO_2_ from baseline until T9 at 2 h (*p* = 0.13). No participant experienced an increase in measured ETCO_2_ up to a value greater than 45 mmHg. No participant experienced an increase in measured ETCO_2_ greater than 6.3 mmHg. The median increase in ETCO_2_ (∆ ETCO_2_) over the study period was 2 mmHg (IQR, 0–1). See Fig. 1.

Pulse Oximetry (SpO_2_%): Sixteen (16 out of 30) participants had a SpO_2_% reading of 100% at baseline, while 14 participants had a value of between 94 and 99% at baseline. Thirteen (13/14) had an increase in SpO_2_% by 5 min on the OxEra^TM^ and one (1/14) had an increase by 20 min.

**Fig. 2:**
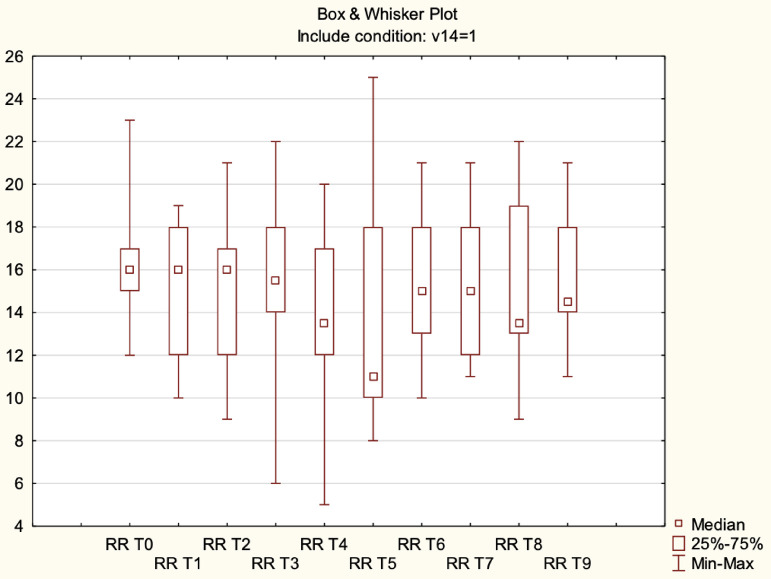
RR from start to the end of the study.

Respiratory Rate (RR. Friedman ANOVA): There were no significant changes in respiratory rate from baseline to 2 h (T9), *p* = 0.32. See Fig. 2. The median RR at T0 was 16 (IQR 14–17), while the median RR at T9 was 16.5 (IQR 13–18).

**Fig. 3:**
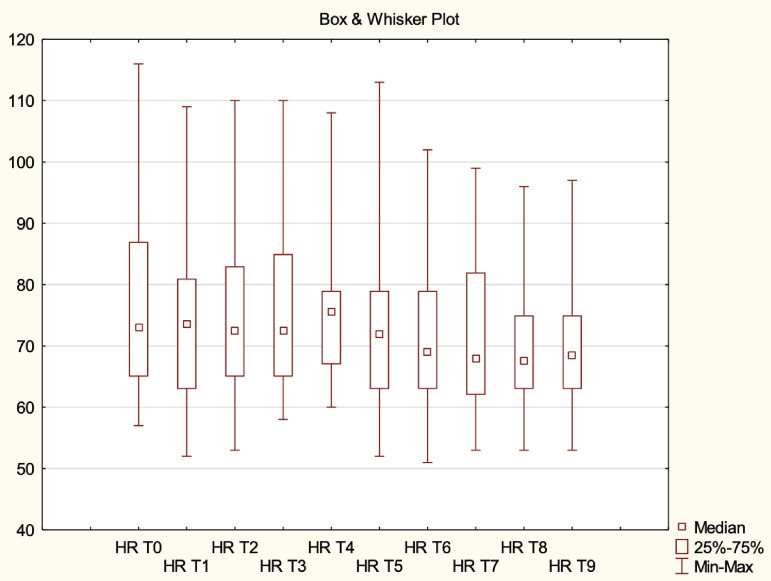
HR from start to the end of the study.

HR: The median HR decreased significantly from 73 (IQR 65–87) at T0, to 68.5 (IQR 63–75) at T9, *p* = 0.000. See Fig. 3.

**Fig. 4:**
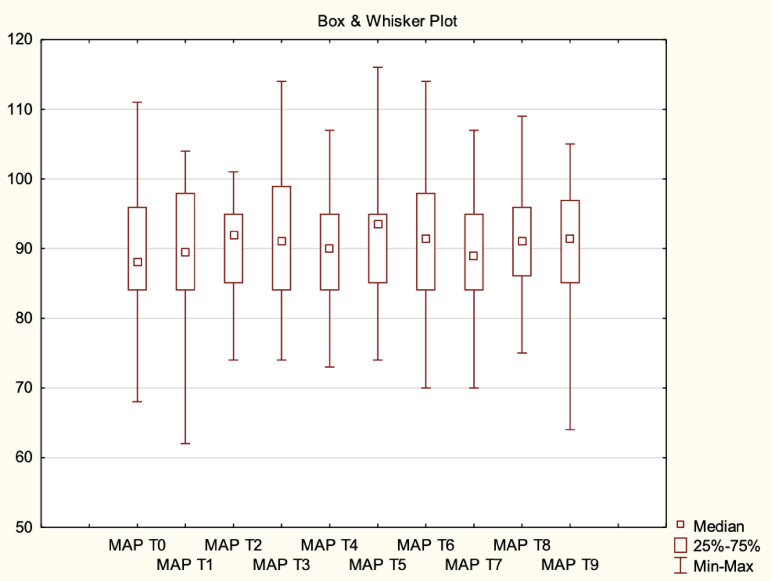
MAP from start to the end of the study.

Mean Arterial Pressure (MAP): There were significant changes in the MAP from baseline to 2 h (T9), *p* = 0.21. See Fig. 4.

**Fig. 5:**
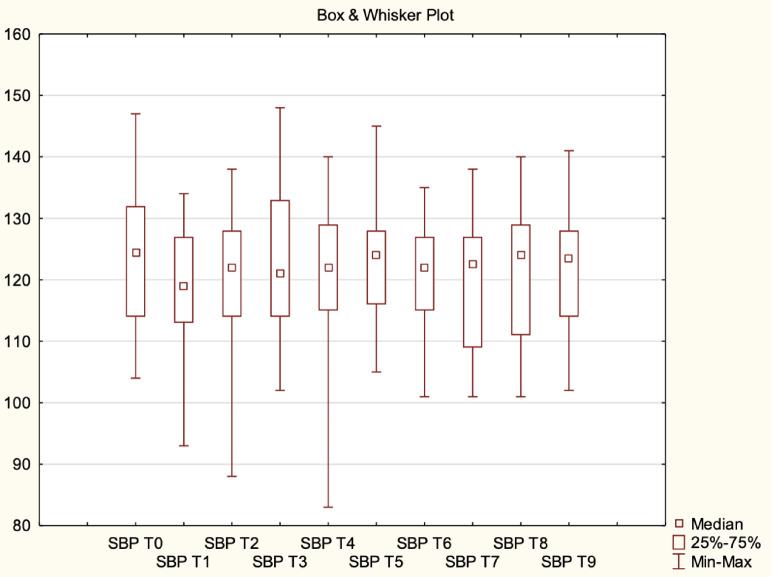
SBP from start to the end of the study.

Systolic Blood Pressure (SBP): There were no significant changes in SBP from baseline to 2 h (T9), *p* = 0.14. See Fig. 5.

**Fig. 6:**
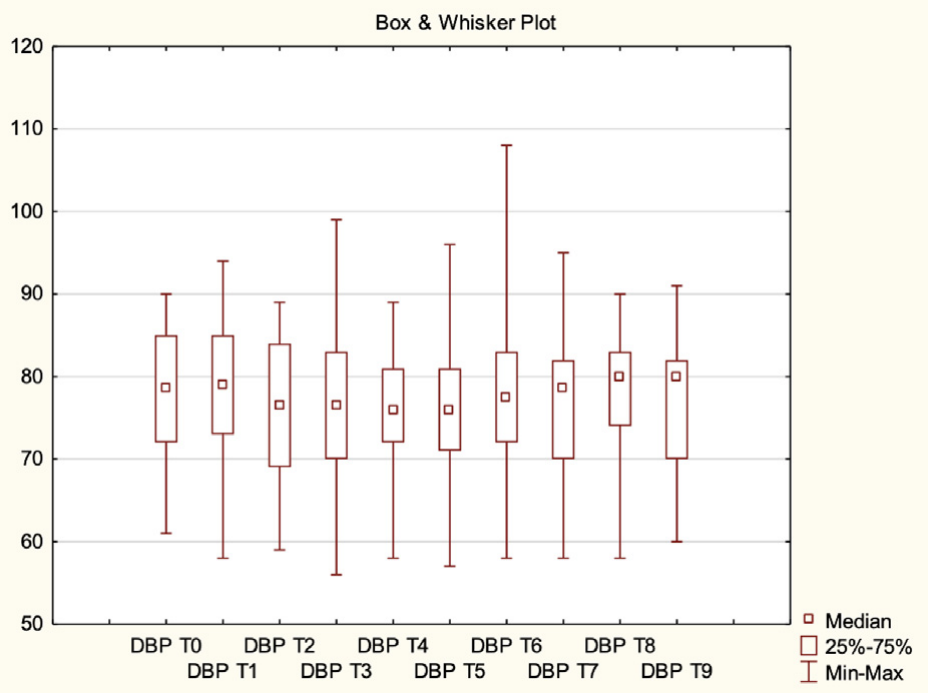
DBP at the start to the end of the study.

Diastolic Blood Pressure (DBP): There were no significant changes in DBP from baseline to 2 h (T9), *p* = 0.33. See Fig. 6.

**Fig. 7:**
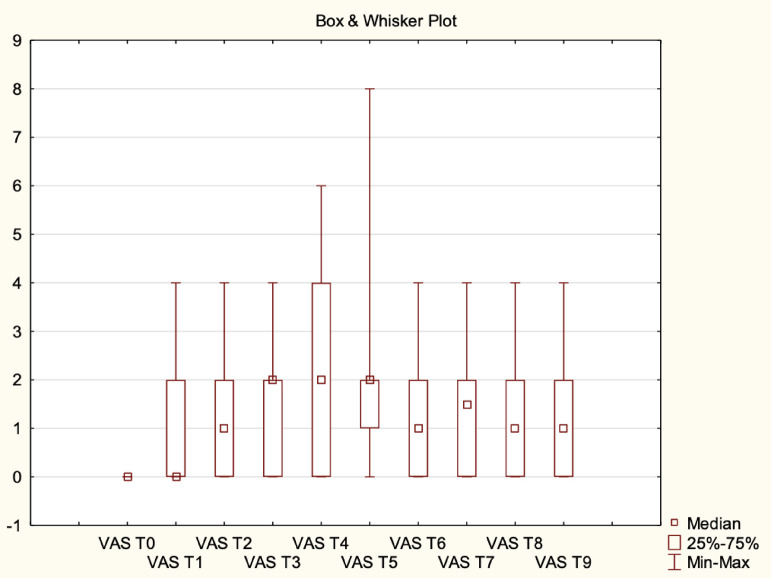
VAS score from start to the end of the study.

VAS Score: The VAS score had a significant increase from T0 to T9, *p* = 0.000. The worst median VAS score was 2 at T3, T4, and T5. This decreased to a median VAS score of 1 by T9. See Fig. 7.

**Fig. 8:**
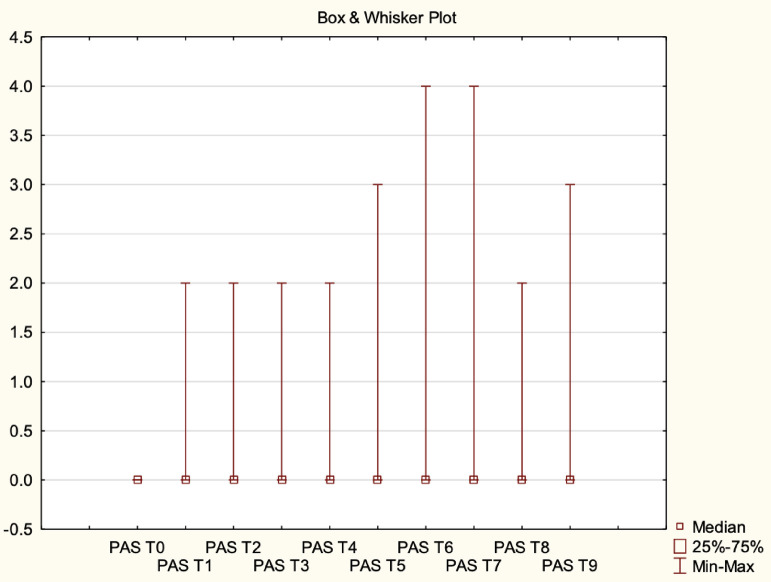
PAS scores from start to the end of the study.

PAS Score: There was no significant increase in PAS scores from T0 to T9, *p* = 0.09. See Fig. 8.

**Fig. 9:**
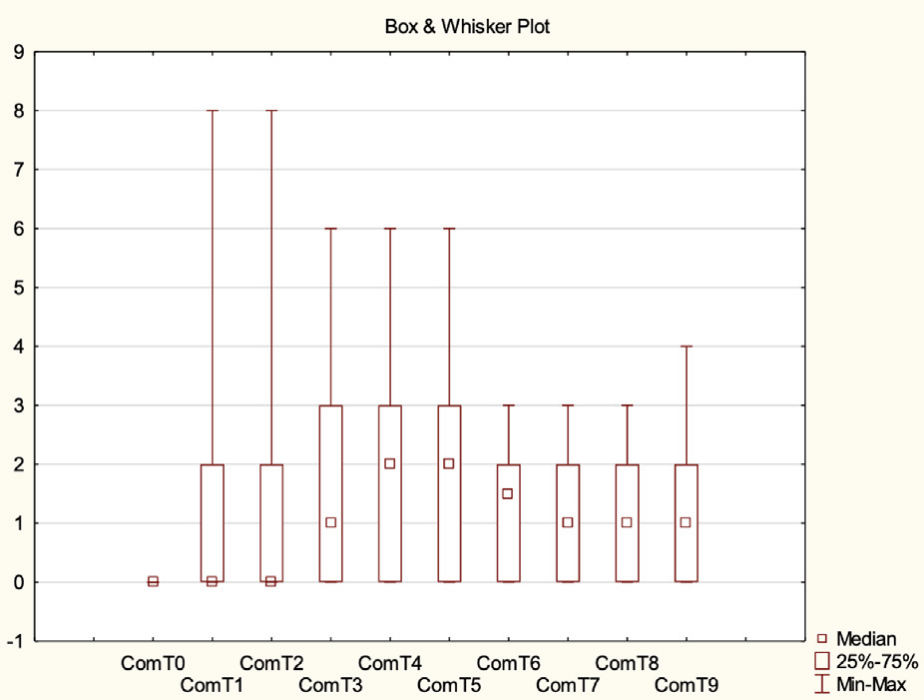
Comfort score from start to the end of the study.

Comfort Score: There was a significant increase in comfort scores from T0 to T9, *p* = 0.000. The worst median comfort score was 2 at T4 and T5 (i.e., the comfort was worst on average at this stage). This decreased to a median comfort score of 1 by T7. See Fig. 9.

## Discussion

Safety: The main aim of this study was to assess the safety of the OxEra^TM^ device concerning the risk of rebreathing. This safety aspect is key amongst many characteristics on which a CPAP system is selected. In our limited resource setting, safety, portability, comfort, ease of use, and the ability to reduce oxygen requirements are key [10]. The risk of rebreathing is a well-established risk and design features suggest the inclusion of a passive/ safety valve [11]. Capnography is a useful tool to detect changes in ETCO_2_ that may be caused by rebreathing [9]. The clinical consequences of exposing patients to rebreathing and increased work of breathing must be avoided, and clinical studies are required to understand these issues [12].

The main finding of our study showed no significant difference in ETCO_2_ from baseline until the end of the study. No participant experienced an increase in ETCO_2_ above the upper reference limit. In addition, no participant experienced an increase in ETCO_2_ greater than the imprecision of the measurement device (capnography devices). This finding confirms the safety of the OxEra^TM^ device concerning rebreathing.

Tolerance of OxEra^TM^ device: Discontinuation of PEP will occur in a significant proportion of patients due to pain and discomfort. A recent study during the SARS CoV-2 pandemic demonstrated that 12% of patients on CPAP discontinued due to pain and discomfort [13]. A CPAP trial showed that 70–80% who were placed on CPAP continued, whereas 5–30% abandoned them. The reasons attributed to abandonment were mainly lacking benefit but also included mask discomfort, anxiety, pain, and noise [14]. In another study, 15% of the participants abandoned CPAP mainly due to its discomfort with the mask [15].

A multi-variable approach to pain and discomfort may be superior to any one method of detection [16]. The normal physiological change in the cardiac system with PEP is a decrease in cardiac output and mean arterial pressure [17].

Respiratory physiological changes of PEP are usually monitored in studies with invasive methods. These studies observed that the work of breathing is shown to be reduced in unhealthy patients with increases in PEP from as little as 5 cmH_2_O [18]. Healthy individuals are not meant to decrease the work of breathing. As expected in our healthy participants, there were no changes in the respiratory rate in the participants in the study. There were no significant changes in mean arterial pressure, systolic blood pressure, or diastolic blood pressure. Similarly, we did not find an increase in heart rate or respiratory rate. The lack of significant changes in these physiological markers suggest tolerance of the OxEra^TM^ device.

Pain/discomfort: Pain and discomfort are notoriously difficult to assess in severe or critically ill patients [17]. Appropriate assessment of pain is key to its management [18]. Validated pain tools like NRS, also known as PAS, and the VAS are required to objectively assess comfort and pain [19]. There was no significant change in the PAS. The changes in the VAS score did not reach the threshold required to initiate acetaminophen or anti-inflammatory drugs, making them unlikely to result in the termination of CPAP therapy [20]. These changes are simply managed by reassurance and observation. The comfort score increased marginally, but once again, it did not reach the threshold for activating a formal pain assessment. These elevations in the VAS and comfort score occurred at positive pressure levels of 20 cmH_2_0. The combination of the lack of physiological changes of pain and the lack of significant indicators of pain using objective scoring systems suggest that the OxEra^TM^ device was well tolerated on a positive pressure as high as 20cmH_2_0, and discontinuation due the pain or discomfort is unlikely.

Limitations: Although we used an imprecision documented in the literature, we did not measure this in the capnograph instrument used for the study. Secondly, we did not measure the positive pressure generated by the OxEra^TM^ device in our subjects; however, this has been previously done for SAHPRA approval. Lastly, we could have improved on the average age of participants. An older population would have distributed the sample size to a greater general population.

## Conclusion

The OxEra^TM^ device is an innovative oxygen delivery device that has many benefits, especially in this era of COVID 19. Currently, it has SAPHRA approval to be used in the COVID 19 pandemic for emergency use.

The OxEra^TM^ device demonstrated safety in terms of risk of rebreathing and was well tolerated up to a positive pressure of 20 cmH_2_0 in this clinical evaluation amongst healthy participants.

With approval for the safety of this device, the OxEra^TM^ may have use in various fields of medicine involving respiratory diseases, specifically in resource and oxygen-limited settings.

## Dissemination of results

The results of this study were presented as a Master of Medicine Report. The report will be displayed on a poster in the Witwatersrand University's School of Medicine, Bienniel Research Day on the 30th of September 2021.

## Author contribution

Authors contributed as follow to the conception or design of the work; the acquisition, analysis, or interpretation of data for the work; and drafting the work or revising it critically for important intellectual content: MTJ contributed 70% and SVB, SO and JB contributed 10% each. All authors approved the version to be published and agreed to be accountable for all aspects of the work.

## Declaration of Competing Interest

The authors declared no conflicts of interest.
